# Multiaxial Fatigue Analysis of Jacket-Type Offshore Wind Turbine Based on Multi-Scale Finite Element Model

**DOI:** 10.3390/ma16124383

**Published:** 2023-06-14

**Authors:** Mengyao Peng, Min Liu, Shuitao Gu, Shidong Nie

**Affiliations:** 1 School of Civil Engineering, Chongqing University, Chongqing 400044, China; pengmy@cqu.edu.cn (M.P.); gust@cqu.edu.cn (S.G.); nieshidong@cqu.edu.cn (S.N.); 2 Chongqing’s Key Laboratory of Structural Wind Engineering and Wind Resource Utilization, Chongqing 400044, China

**Keywords:** jacket, multi-scale model, multiaxial fatigue damage, local joint

## Abstract

The fatigue damage of a local joint is the key factor accounting for the structural failure of a jacket-type offshore wind turbine. Meanwhile, the structure experiences a complex multiaxial stress state under wind and wave random loading. This paper aims to develop a multi-scale modeling method for a jacket-type offshore wind turbine, in which local joints of the jacket are modeled in a detail by using solid elements, and other components are modeled via the common beam element. Considering the multiaxial stress state of the local joint, multi-axial fatigue damage analysis based on the multiaxial *S–N* curve is performed using equivalent Mises and Lemaitre methods. The uniaxial fatigue damage data of the jacket model calculated using the multi-scale finite element model are compared with those of the conventional beam model. The results show that the tubular joint of jacket leg and brace connections can be modeled using the multi-scale method, since the uniaxial fatigue damage degree can reach a 15% difference. The comparison of uniaxial and multiaxial fatigue results obtained using the multi-scale finite element model shows that the difference can be about 15% larger. It is suggested that the multi-scale finite element model should be used for better accuracy in the multiaxial fatigue analysis of the jacket-type offshore wind turbine under wind and wave random loading.

## 1. Introduction

Wind energy is the fastest growing renewable energy source in the world, and offshore wind power has a high potential for development [1,2,3]. Jacket-type support structures are an important part of offshore wind turbines [4], and the tubular joints are susceptible to fatigue damage under the combined effects of wave and wind loads [5].

There are usually two approaches to the structural analysis of jacket-type offshore wind turbines. One is the global finite element modeling to analyze from macroscopic scale [6,7]. The joints of global model are established using beam elements, ignoring the influence of nodal effects, such as the flexibility, geometry and weld configuration of its substructure joints [8,9]. The other is local modeling via small-scale analysis, and the local joint flexibilities of tubular K/DK-joints [10,11], T/Y-joints [12,13,14], or X-joints [15,16] are established individually using solid or shell elements for analysis. For offshore structures subjected to typical wave and wind loading, local joints are difficult to determine the boundary conditions, ignoring the coordination between the global model and local model. 

Multi-scale finite element simulation can provide a better solution in this regard. Small-scale solid elements are used to simulate local joints, while large-scale beam elements are used to simulate other components in the structure. This multi-scale model can capture not only the global structural behavior in terms of displacements and accelerations, but also the local nodal behavior in terms of stresses and strains without huge computational costs. Wang et al. [17] developed a multi-scale model to analyze the failure analysis of transmission tower structures under the action of down-strike storm currents. Zhu et al. [18] applied the multi-scale model to the structural health monitoring of cable-stayed bridges. Fang et al. [19] established a multi-scale finite element model for the fatigue assessment of a welded beam-to-column connection in a steel tall building. Jan Dubois et al. [20] took advantage of an accurate description of local joint behaviors using super-elements, enabling more accurate load simulations. The key issue of multi-scale finite element simulation is the coupling of different scale elements, e.g., the beam, shell and solid, at the interface. Several researchers have provided various coupling constraint methods, such as the Lagrange multiplier method, penalty method, transition element method and multi-point constraint method [21,22].

Jacket-type offshore wind turbines are subjected to complex environmental loads, and the joint fatigue problem is prominent under wind and wave loads. Some scholars have carried out fatigue analysis work on the tubular joints of jacket structures, but only a few studies has been performed on the multiaxial fatigue behavior of offshore wind turbines [23,24,25,26]. However, due to the complexity of the local shape of the structure, multiple external input loads and other factors, the K-type tubular joints of the jacket-type offshore wind turbine structure is often in a multiaxial stress state during service, which is actually a multiaxial fatigue problem [27,28]. Multiaxial fatigue analysis mainly includes two methods, one is based on the static strength theory method, and the other is the critical plane method. The von Mises equivalent stress method based on the static strength theory is commonly used in the engineering field [29,30,31], but it ignores the effect of hydrostatic pressure, which has been shown to have an important effect on fatigue [32,33]. Ge et al. [34] proposed an improved Lemaitre equivalent multiaxial stochastic fatigue estimation method. The method is used to consider the effect of hydrostatic pressure based on stress invariants and also to introduce a multiaxial *S–N* curve to predict the multiaxial fatigue damage of structures.

As can be seen in this review of existing literature, only a few studies have been performed on the multiaxial fatigue behavior of jacket-type offshore wind turbines, and most have dealt with the conventional beam elements model. Although the multi-scale method is used in a variety of large-scale structural calculations, its application to the structural analysis of jacket-type offshore wind turbines is still lacking. The offshore wind turbines are subjected to aerodynamic loads based on the quasi-steady blade element momentum theory and the hydrodynamic loads based on the Morrison equation, resulting in complex stress states at local joints. Therefore, multi-scale finite element models are urgently needed for structural dynamic analysis, and multiaxial fatigue methods need to be considered for the fatigue damage assessment of tubular joints of the jacket. Via global and local cooperative analyses, the accuracy is improved without increasing the computational effort, which provides a new idea for engineering pre-designs. 

In this study, the multi-scale method is employed to jacket-type offshore wind turbine finite element models, and multi-point constraint equations are used for the interface coupling of different scale elements. To verify the multi-scale finite element model, modal analysis is performed. The fatigue damage results of multi-scale model and beam model are evaluated. Multiaxial fatigue behavior of a jacket-type offshore wind turbine under wind and wave random loading was estimated, with particular attention being paid to the multi-scale model of a typical K-type tubular joint. The results of uniaxial and multiaxial fatigue damage are compared.

## 2. Reference Wind Turbine Model

**Figure 1:**
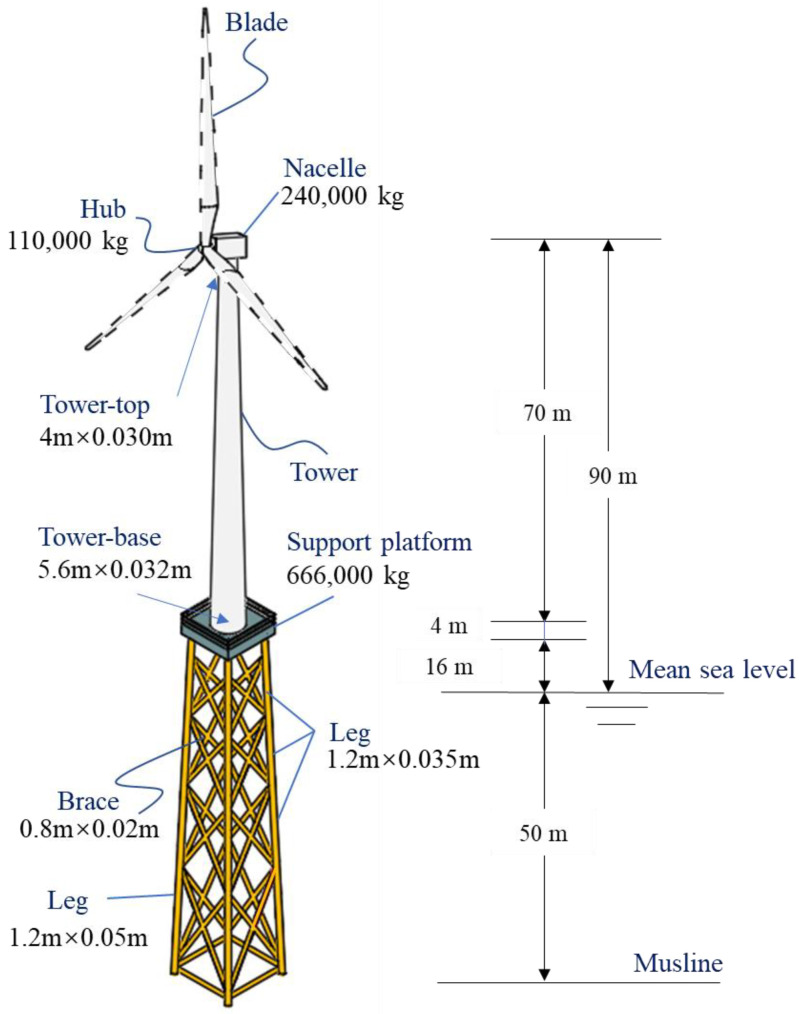
The reference model of a 5-MW NREL wind turbine.

The reference model of a 5-MW National Renewable Energy Laboratory (NREL) wind turbine [35] is used for examples in many related studies, as shown in Figure 1. The structure comprises the hub and nacelle, the tower, the support platform and the jacket-type foundation, whose major geometric and material parameters are given in Table 1 and Table 2, respectively. More details can be found in [36].

### 2.1. Multi-Scale Modeling of the Jacket-Type Offshore Wind Turbine

The multi-scale finite element model of the jacket-type offshore wind turbine described above was built using commercial finite element software, ANSYS. Multi-scale finite element modeling requires the delineation of potential local failure regions and other parts of the global model, including the following three steps: the first step is to refine the local model using solid elements for the typical joint, the second step is to construct a global model using beam elements, and the third step is to couple the global and local models using constraint equations to integrate the multi-scale model.

**Figure 2:**
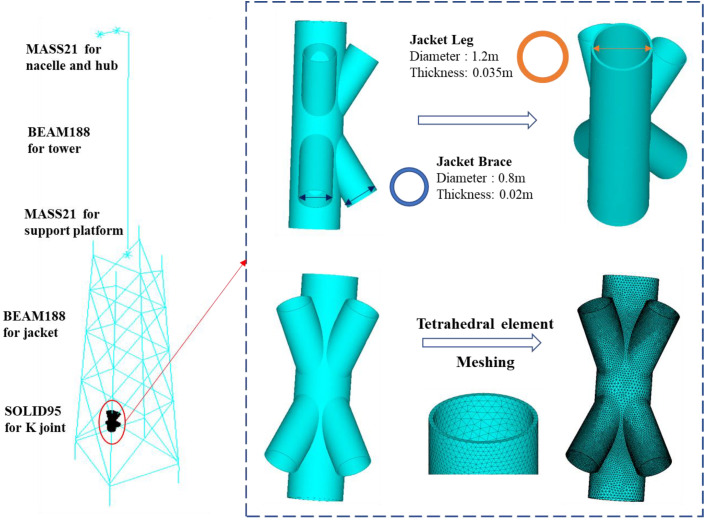
Multi-scale model of jacket-type offshore wind turbine.

Firstly, a local model was established to determine the fatigue failure control point of the structure. For the sake of a clear demonstration, only one typical and most important joint was selected to construct a detailed local model. Referring to the work by Wei and Yuan [37], it is generally considered that the most severe plastic damage occurs in the third and fourth X frame levels of the jacket. Therefore, the K-type tubular joint, as shown in Figure 2, was selected as the fatigue control location for fatigue damage analysis. Higher-order SOLID95 elements, which can simulate irregular shapes with no loss in accuracy, were used to model the selected joint.

After local modeling, global modeling was performed by using BEAM188 elements to simulate all other components. Different from the conventional beam elements model, the global model of multi-scale finite element model has a blank space in the local joint position. The literature shows that the effect of brace length on the stiffness of the multi-scale model is negligible when the model length is about 2.5 times larger than the brace diameter [20]. In addition, the nacelle, hub and support platform were reduced to mass points using MASS21 elements, respectively. Finally, in order to couple the global beam model and the local solid model, multi-point constraint equations were derived based on the virtual work principle to achieve a displacement (strain)-compatible and force (stress) equilibrium at the coupled interface. The displacement constraint equations for coupling the beam and solid models are given in [38]. 

**Figure 3:**
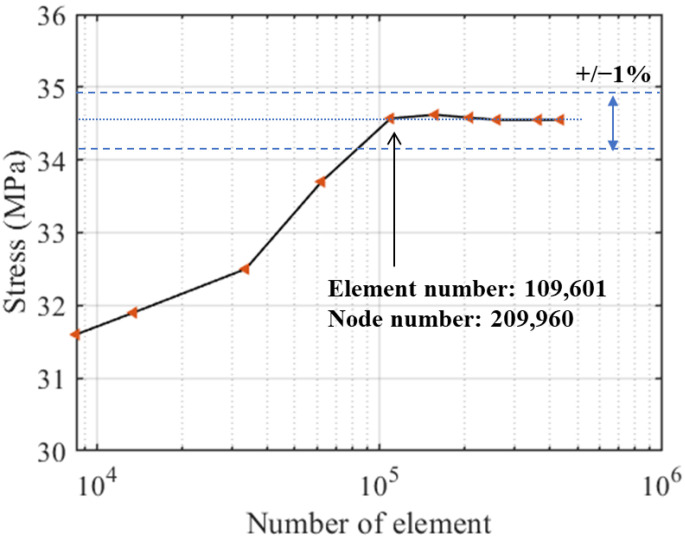
Sensitivity analysis to different number of elements.

In order to perform mesh sensitivity analysis, several models of the local K-type tubular joint with different number of elements were analyzed. The results in Figure 3 show that meshes with approximate 109,601 elements are both behaviorally and computationally efficient for the K-type tubular joint using tetrahedral element shapes. The beam element size was meshed to 0.5 m according to [36]. Therefore, the multi-scale finite element model of the jacket wind turbine has 1942 beam elements, 109,601 solid elements and a total of 213,797 nodes.

### 2.2. Multi-Point Constraints

Multi-scale interface coupling is the key to establishing a multi-scale model of the structure. In the multi-scale model of a jacket-type offshore wind turbine, the coupling between beam element and solid element needs to be considered. Due to the different number of element degrees of freedom, the nodes of beam element have six directional degrees of freedom (three translational displacement ones, *U_x_*, *U_y_* and *U_z_*, and three rotational ones, *R_x_*, *R_y_* and *R_z_*), and the nodes of the solid element have three directional translational degrees of freedom, *U_x_*, *U_y_* and *U_z_*. Therefore, the multi-scale model of an offshore wind turbine needs to consider the coupling of degrees of freedom and implement it in the finite element solution.

**Figure 4:**
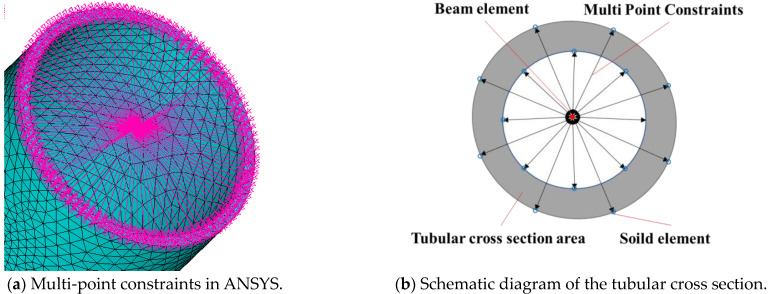
Multi-point constraints connection between beam and solid element.

Multi-scale model interface coupling is achieved by means of multi-point constraint equations, as shown in Figure 4. The multi-point constraint method is considered to be an attractive coupling method based on the force and displacement constraint equations used to couple elements of different scales. The constraint equations for the interface coupling are expressed as the nodal displacements of the beam elements equal to the generalized displacements at the interfaces of the solid elements as follows:

**c(u_b_,u_s_)** = **u_b_** − **Cu_s_** = **0** (1)

where **u_b_** and **u_s_** denote the nodal displacement vectors of the beam model and solid model interfaces, respectively, and **C** is the constraint equation coefficient matrix.

### 2.3. Modal Analysis and Validation via Natural Frequency Comparison

In order to verify the accuracy of the multi-scale finite element model of the jacket-type offshore wind turbine structure, modal analysis was performed and compared with the results calculated using the BeamDyn module of FAST. The BeamDyn module uses the finite element for modeling, and is considered to be the most professional simulation and analysis software for wind turbine calculation at present [30]. The structural models were established in three different ways, i.e., the beam element model was established using FAST software, and the simplified beam model and multi-scale model were established using commercial finite element software. The modal analysis takes FAST calculation results as the standard and analyzes the relative errors of simplified beam model and multi-scale model using commercial finite element software ANSYS. Damping has significant influence on the dynamic response of the jacket-type offshore wind turbine. The Rayleigh damping with a damping ratio of 1% is applied to all modes in this study [35].

The results of the first eight-order modal vibration model calculation comparison are shown in Table 3. The lower-order modes of three finite element models are basically the same, and the maximum relative errors of 8.98% and 8.74% appear in the higher-order modes of the left and right bending of the structure, respectively. The reason for this analysis is that the low-order vibration shape indicates the overall characteristics of the structure, and the high-order vibration shape reflects the local characteristics of the structure, and there are slight differences between the local locations of the FAST model and the commercial software beam model. Meanwhile, the results show that the free vibration frequencies of the multi-scale model and the beam model are in good agreement with the difference of less than 9%. 

**Figure 5:**
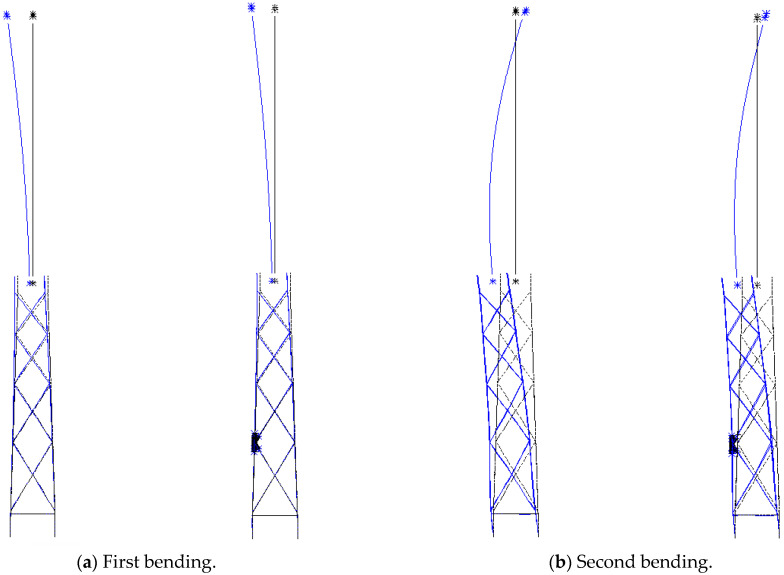

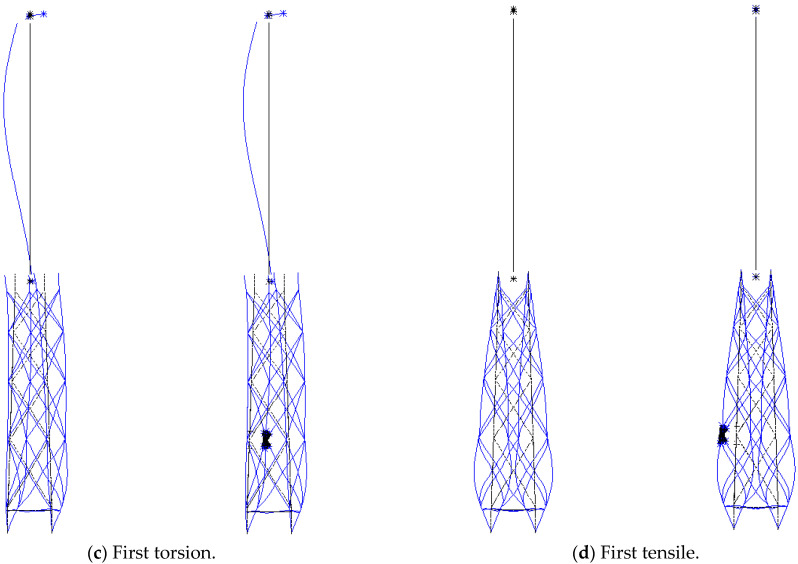
Modal analysis: beam model (**left**), multi-scale model (**right**), undeformed (**black line**) and deformed (**blue line**).

Figure 5 provide a comparison between the first bending, second bending, first torsion and tensile modal shape as calculated by ANSYS. Starting with the torsional modal shape, the deformation is dominated by the local diagonal bracing members of the jacket-type offshore wind turbine structure. The multi-scale finite element model is basically consistent with the beam model for each order mode shape.

**Figure 6:**
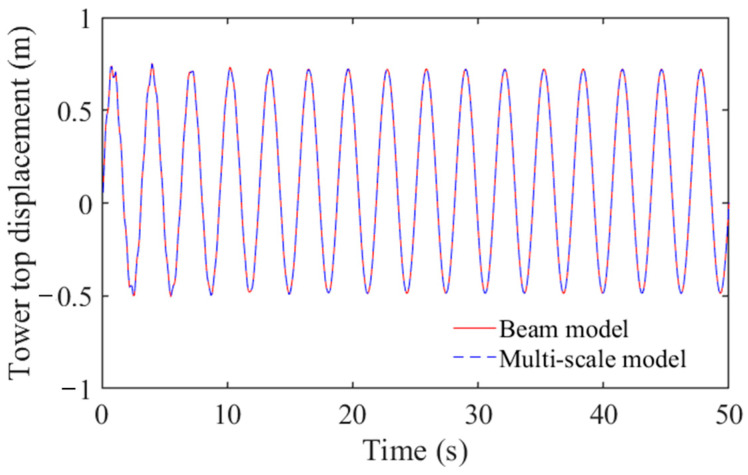
Time history of tower top displacement under forced analysis.

To include the effect of damping, forced vibration analysis was performed. The harmonic load with excitation amplitude of 2000 KN and a frequency of 0.32 Hz was given at the top of the tower. The results in Figure 6 show that the displacement responses at the top of the tower under forced vibration in both models match well. Via modal analysis and forced vibration analysis, the multiscale model was verified for subsequent numerical research.

## 3. Multiaxial Random Fatigue Analysis Methodology

Multiaxial fatigue damage analysis methods based on a multiaxial *S–N* curve were used in this study. The calculation steps can be divided into four major parts. The first step was to consider the stresses required to estimate the fatigue damage as multi-axial equivalent stresses. Then, a suitable multi-axial cycle counting method was selected, such as the rainflow-counting method. Next, the multi-axial *S–N* curve, considering the effect of shear stress, was defined. Finally, the fatigue damage accumulation theory was applied to the jacket-type offshore wind turbine structure.

### 3.1. The von Mises Equivalent Stress

The von Mises equivalent stress method for multiaxial random fatigue proposed by Pitoiset and Preumont [29] is based on extensions of the static yield theory. It is supposed that the fatigue life under multiaxial loading can be predicted via calculating the von Mises stress on which the classical uniaxial stochastic fatigue model can be applied [34].

The von Mises stress equivalent, $s_{eq}$, is defined by the quadratic relationship:

(2) $$s_{eq}^{2}=s_{xx}^{2}+s_{yy}^{2}+s_{zz}^{2}-s_{xx}s_{yy}-s_{xx}s_{zz}-s_{yy}s_{zz}+3s_{xy}^{2}+3s_{xz}^{2}+3s_{yz}^{2}$$

where $s_{xx}$, $s_{yy}$ and $s_{zz}$ and $s_{xy}$, $s_{xz}$ and $s_{yz}$ are the normal and shear stresses, respectively. 

### 3.2. The Lemaitre Equivalent Stress

The influence of hydrostatic stress is ignored in the definition of the von Mises equivalent stress, which has a significant effect on fatigue and fracture. Lemaitre equivalent stress can be a good solution to this problem, which is defined by Lemaitre and Desmorat [39] in damage mechanics as:

(3) $$s_{eq}^{*}=s_{eq}{\left[\frac{2}{3}(1+v)+3(1-2v){\left(\frac{s_{m}}{s_{eq}}\right)}^{2}\right]}^{\frac{1}{2}}$$

where v is the Poisson’s ratio of the material, $s_{eq}^{*}$ is the Lemaitre equivalent stress, and the hydrostatic stress, $s_{m}$, can be expressed as:

(4) $$s_{m}=\frac{s_{xx}+s_{yy}+s_{zz}}{3}$$

### 3.3. The Multiaxial S–N Curve

**Figure 7:**
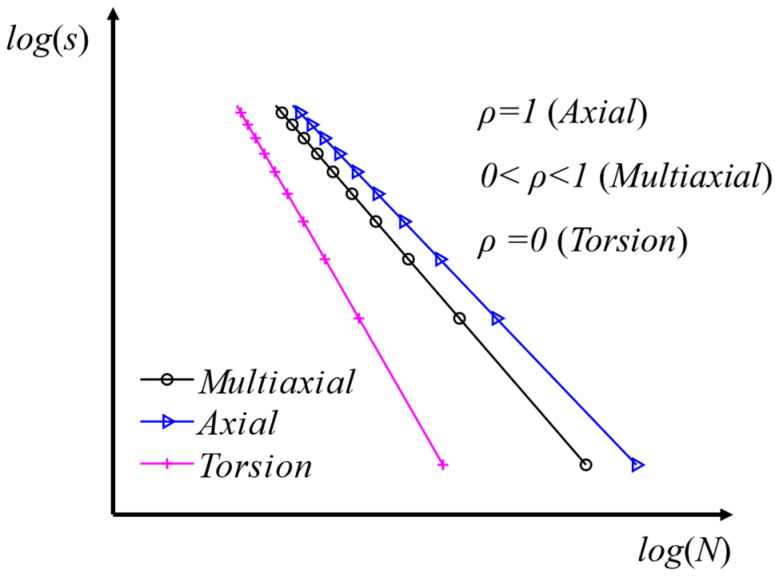
Multiaxial *S–N* curve [34].

This paper assumes that the material fatigue life under multiaxial cyclic loading can be summarized using a multiaxial *S–N* curve [34], which shows a linear relation in log–log charts between the equivalent multiaxial stress amplitude and the number of cycles to failure, as shown in Figure 7.

The multiaxial *S–N* curve can be obtained according to fully reversed uniaxial and torsional loading, and a parameter similar to triaxial stress ratio is defined:

(5) $$\rho =\frac{3\bar{\sigma_{m,a}}}{\bar{\sigma_{eq,a}^{*}}}$$

where $\bar{\sigma_{m,a}}$ is the mean value of the hydrostatic stress amplitude; $\bar{\sigma_{eq,a}^{*}}$ is the mean value of the equivalent stress amplitude, which can be obtained by counting the time history of the stress response using the rainflow-counting method.

The multiaxial *S–N* curve can be estimated from the calculated, ρ, as a linear combination of the *S–N* curves for uniaxial tension–compression and torsion [28]:

(6) $$k_{app}=k_{tor}+\rho (k_{axi}-k_{tor})$$

(7) $$\log(C_{app})=\log\left({\left(\sqrt{2(1+v)}\right)}^{k_{tor}}C_{tor}\right)+\rho \left(\log(C_{axi})-\log\left({\left(\sqrt{2(1+v)}\right)}^{k_{tor}}C_{tor}\right)\right)$$

where *C* and *k* are the material constant and the slope of the *S–N* curve. The subscripts ‘*axi*, *tor*, *app*’ indicate the corresponding axial, torsional and multi-axial *S–N* curves, respectively. Thus, the power expression for the multiaxial fatigue *S–N* curve can be written as:

(8) $$N=C_{app}S^{-k_{app}}$$

It can be seen that when, ρ=1 the multiaxial fatigue *S–N* curve fully satisfies the uniaxial tension–compression *S–N* curve; when ρ=0, it also fully satisfies the *S–N* curve for pure torsion. The basic fatigue constants for the joint of the jacket-type offshore wind turbine suggested by DNV-RP-C203 [40] are given in Table 4. It should be noted that the material parameters related to axial fatigue in this paper are assumed to be $\sqrt{3}$ times larger than the pure torsional fatigue [34]. 

Multiaxial fatigue damage is evaluated via a multiaxial *S–N* curve and the rainflow-counting method. The stress ratio of all cycles are first corrected to −1 using the goodman formulation, and then counted to obtain the stress cycle range, *S*, and number of cycles, *n,* which determine the fatigue damage *D*. The fatigue damage rate, *E*[*D*], can be express as:

(9) $$D=\sum_{i=1}^{l}d_{i}=\sum_{i=1}^{l}\frac{n_{i}}{N_{i}}=\sum_{i=1}^{l}\frac{n_{i}}{C_{app}S_{i}^{-k_{app}}},\;E[D]=\frac{D}{T}$$

where *l* is the number of stress blocks and *N_i_* is the cycles calculated via the multiaxial *S–N* curve, *T* is the time length and *E*[*D*] represents the fatigue damage per unit time.

## 4. Results and Discussion

In this section, taking the 50-year wind and wave load condition as a representative case, the fatigue damage of the jacket-type offshore wind turbine typical K-type tubular joint is discussed using the structural model and the fatigue analysis method.

### 4.1. Load Case and Representative Fatigue Control Points

**Figure 8:**
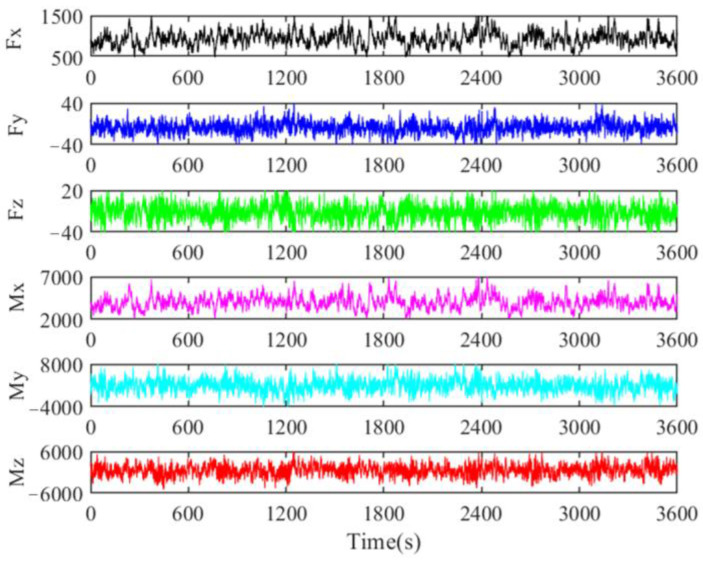
Wind load time history in six directions on the rotor obtained using the FAST tool.

In this study, jacket-type offshore wind turbines were subjected to wind and wave environmental loads. The wind loads were obtained by calculating the lift and drag forces on the blades from the incoming wind speed. To facilitate the dynamic analysis of the offshore wind turbine support structure, the lift and drag loads on the blades were modeled as equivalent loads acting at the hub center, including three forces and three moments, as shown in Figure 8. The wave loads were obtained via calculating the drag and lift forces on the base structure of the jacket through the significant wave height and spectral peak period. In this study, the corresponding wind and wave loads were obtained using FAST software. 

**Figure 9:**
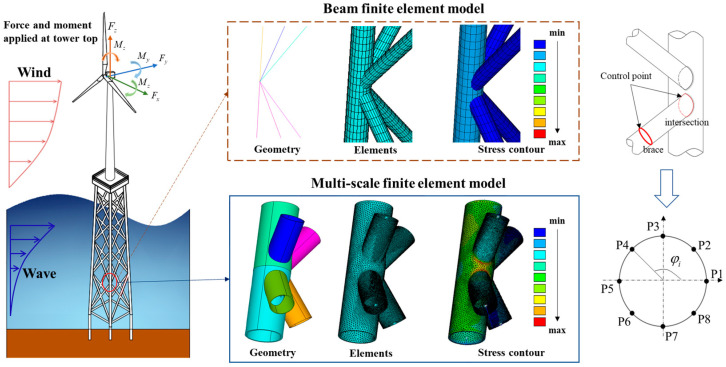
The typical K-type tubular joint in the beam model and multi-scale model.

The fatigue analysis conditions were determined via the wind and wave random loading of the mean return period of 50 years [41], and detailed parameters of the calculations are shown in Table 5. The fatigue damage control location was selected at the intersection of the jacket leg and diagonal brace in a typical K-type tubular joint of the jacket substructure. To supplement the advantages of the multi-scale model, the location of the diagonal brace end point of the K-type tubular joint solid element model was used as the comparison analysis point. For the beam model, eight representative points (referred to as P1 to P8) evenly distributed along the cross-section were chosen for fatigue damage estimation. Schematic diagrams of typical K-type tubular joint and the representative points in the beam and multiscale models are illustrated in Figure 9. The resultant stresses of these chosen points are determined as:

(10) $$s_{xx,i}=\frac{N_{x}}{A}+\frac{M_{x}}{I_{x}}r\sin\varphi_{i}-\frac{M_{y}}{I_{y}}r\cos\varphi_{i}$$

where *N**_x_*** is the axial force; *A* is the nominal cross section area; *r* is the radius of the cross-section; $\varphi_{i}$ is the angle between the point and the *x*-axis of the cross section; *M_x_* and *M_y_* are the bending moments; *I_x_* and *I_y_* are the sectional moments of area. For the multi-scale finite element model, the stress components of fatigue control location can be obtained directly using commercial software, ANSYS. 

### 4.2. Uniaxial Fatigue Comparison between Multi-Scale Model and Beam Model

#### 4.2.1. Fatigue Results of the Jacket at the Intersection between Leg and Brace

The stress power spectral density and time history of a typical joint at the intersection between the leg and brace was calculated using full time domain analysis considering wind and wave loading. The results of uniaxial stress states for two models including beam model and multi-scale finite element model are discussed for the maximum fatigue damage point among the eight points at the control location. 

**Figure 10:**
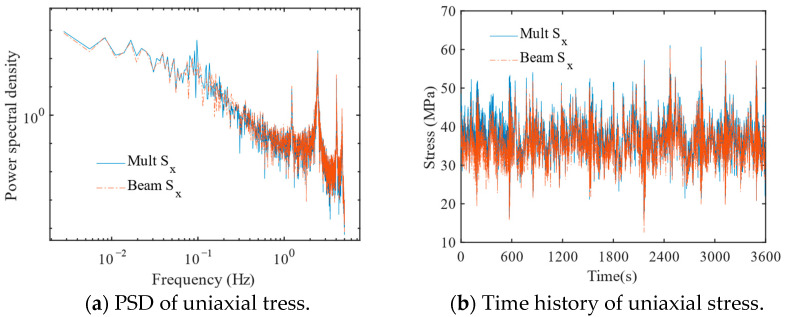
Stress response at the intersection between leg and brace.

Figure 10a gives the power spectral density of the beam model and the multi-scale finite element model. It is deviated in the low frequency part, where the peak at the low frequency corresponds to the wave spectrum peak frequency. The results show that accurate modeling of the K-type tubular joint enables the response at low frequencies to be fully captured. Figure 10b shows the comparison of the stress time histories of two models. The stress response of the multi-scale finite element model is slightly larger than that of the beam model, which may be due to the contribution of the higher-order modal shapes to the response.

The response characteristics (mean value, $s_{m}$, variance, $\sigma_{x}$, skewness, $s_{k}$, and kurtosis, $K_{u}$) and fatigue damage rate, *E*[*D*], results for the multi-scale and beam models at the fatigue failure control points are given in Table 6, respectively. The main differences are reflected in the skewness and variance, and the difference in standard deviation will directly affect the fatigue damage results between the multi-scale model and the beam model after the power scaling of the material parameters of the *S–N* curve. This results in a difference of about 15% in fatigue damage rate between the two models.

#### 4.2.2. Fatigue Results of the Jacket at the Brace

**Figure 11:**
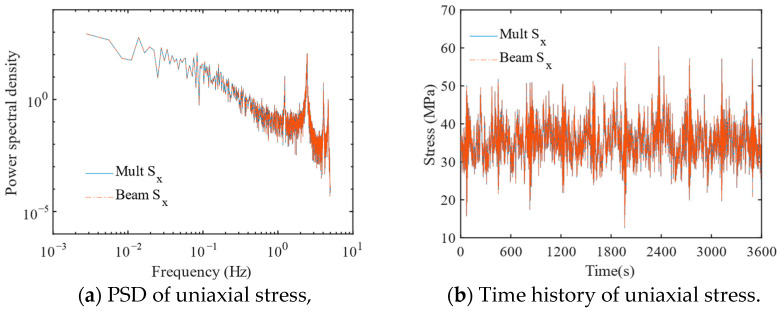
Uniaxial stress response at the brace.

The stress responses of the jacket K-type tubular joint at the brace for the multi-scale finite element model and the beam model was also calculated. Figure 11 shows that the stress response power spectral density and time histories of both models are well matched, and the results are generally consistent. It indicates that for non-concerning regions such as the brace location, beam element simulation is sufficient. In contrast, the multi-scale modeling method has a significant impact on the fatigue results at the intersection locations of typical K-type tubular joint, and the concern region requires smaller-scale elements to be accurately modeled for fatigue analysis. 

### 4.3. Multi-Scale Model Comparison of Uniaxial and Multiaxial Fatigue Methods

**Figure 12:**
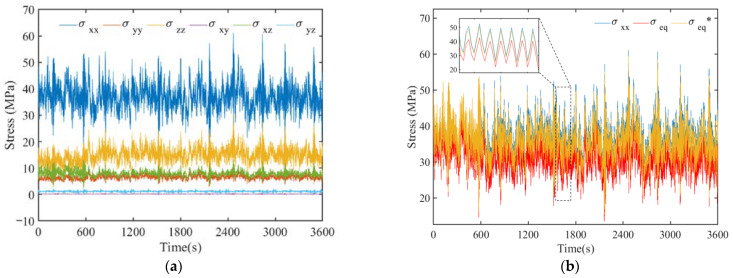
Stress time histories of multi-scale model at P2. (**a**) Time history of normal and shear stresses in three directions. (**b**) Time history of normal, von Mises and Lemaitre stress.

In this subsection, representative points on the intersection of typical K-type tubular joint between jacket leg and diagonal brace in the multi-scale finite element model were selected as fatigue damage control points for the multiaxial stress response discussion. The stress time histories of the control point, P2, as an example are shown in Figure 12. Figure 12a includes the normal and shear stresses in three directions (i.e., $s_{xx}$, $s_{yy}$ and $s_{zz}$ and $s_{xy}$, $s_{xz}$ and $s_{yz}$), obtained via transient analysis. Figure 12b gives the time histories of the normal stresses in the *x*-axis, von Mises and Lemaitre equivalent stresses, and the latter two were obtained using Equations (2) and (3).

**Figure 13:**
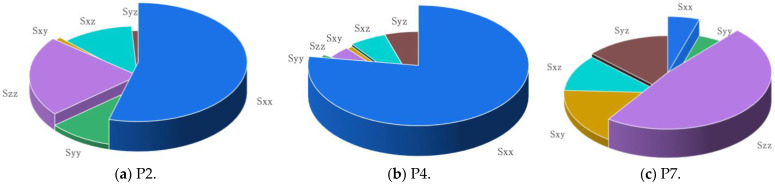
The proportion of each stress component at representative points.

Figure 13 shows the proportions of the mean values of the six stress components at the three representative points, and the thickness of the pie chart represents the variance of the stress time history. It can be found that Figure 13a is dominated by normal stress in the x-direction, which is also influenced by normal stress in the z-direction and the shear stress in the *xz*-direction. Figure 13b is also dominated by normal stress in x-direction, and the stress components in other directions are almost negligible. In Figure 13c, the normal stress in the z-direction is dominant, and the effect of shear stress is significant. For a typical K-type tubular joint with complex environmental loading, it is inaccurate to simplify the representation of the uniaxial stress state in the x-direction only. Due to the influence of other stress components, the multiaxial stress state of the typical K-type tubular joint cannot be neglected.

For further inspection of the multiaxial fatigue analysis data, the comparison results of uniaxial stress, multiaxial von Mises equivalent stress and Lemaitre equivalent stress fatigue damage at the three representative points are presented in Table 7, Table 8 and Table 9, respectively. The results in Table 7 show that the difference between the multi-axis von Mises equivalent stress and uniaxial stress fatigue damage results is about 10%, while the difference between the Lemaitre equivalent stress considering the effect of hydrostatic pressure is 14%. This is because the representative P2 uniaxial stress plays a relatively dominant role, and the hydrostatic pressure is larger. From Table 8, it can be seen that the difference between multiaxial von Mises equivalent stress and uniaxial stress is small because uniaxial stress plays an absolutely dominant role at representative P4. The difference between the multiaxial Lemaitre equivalent results is about 13%, and the effect of hydrostatic pressure is not negligible. The results in Table 9 further illustrate that multiaxial fatigue analysis had a significant impact on the damage results, with a difference from 70% to 80% compared to that of uniaxial fatigue. Due to the non-dominant role of uniaxial stress at representative P7, the shear stress component accounts for a relatively large amount. In addition, the introduced correction of multi-axis *S–N* curve considering the effect of shear stress also has an impact on the multi-axis fatigue damage results. The above results show that for offshore wind turbine jacket-type substructure, multiaxial fatigue analysis is essential.

## 5. Conclusions

In this paper, multiaxial fatigue damage analysis based on a multi-scale finite element model is applied to jacket-type offshore wind turbines under wind and wave loads. The typical K-type tubular joint of jacket considering local features are simulated using small-scale solid elements, and other components are simulated using beam elements. The results of the multi-scale model compared with the beam model show that the point uniaxial fatigue damage at the intersection of the conduit leg and diagonal brace differs by 15%, and the point damage at the diagonal brace is well matched. The reason for this is that the multi-scale model considers the connection characteristics and local geometric features of the K-type tubular joint, and the effect of higher-order modal shape on the statistical properties of the dynamic time history (e.g., skewness and variance). It implies that the multi-scale finite element model is essential for fatigue damage analysis applied at typical joints, while the accuracy of the beam model is sufficient for non-concerning regions of other members.

In addition, multiaxial fatigue damage based on a multi-scale finite element model was analyzed. Multiaxial *S–N* curves were introduced using two equivalent stresses (i.e., Mises and Lemaitre) methods for multiaxial fatigue damage analysis. Based on the multi-scale finite element model, the uniaxial and multiaxial fatigue damage was compared at three representative points. The results show that compared with uniaxial fatigue damage, the difference of multiaxial von Mises equivalent method is about 10%, and the difference of multiaxial Lemaitre equivalent method considering hydrostatic pressure is about 14%. Especially when the uniaxial stress component is dominant, the difference between uniaxial and multiaxial fatigue damage is reduced to 6~13%. However, when other stress components dominate, the difference of multiaxial fatigue damage results reaches 70~80%, and multiaxial stress state is not negligible. It is suggested that the multi-scale finite element model should be applied to typical joints of jacket-type offshore wind turbines under wind and wave random loading for multiaxial fatigue analysis to improve the accuracy of analysis.

## Figures and Tables

**Table 1:** Geometric parameter values of jacket-type offshore wind turbine.

Geometric Parameter	Value
Support platform height (m)	16
Support platform volume (m^3^)	4 × 9.6 × 9.6
Turbine hub height (m)	90
Tower length (m)	68
Tower-base outer diameter (OD) (m)	5.6
Tower-base thickness (m)	0.032
Tower-top OD (m)	4
Tower-top thickness (m)	0.03
Jacket leg OD (m)	1.2
Jacket leg thickness (m)	0.05 (4th level) − 0.035 (1st to 3rd level)
Brace OD (m)	0.8
Brace thickness (m)	0.02
Pile OD (m)	2.082
Pile thickness (m)	0.6

**Table 2:** Material parameter values of jacket-type offshore wind turbine.

Geometric Parameter	Value
Young’s modulus (Pa)	2.1 × 10^11^
Density (kg/m^3^)	7850
Poisson’s ratio (m)	0.33
Support platform mass (kg)	666,000
Hub mass (kg)	110,000
Nacelle mass (kg)	240,000

**Table 3:** Modal analysis results of multi-scale model and beam model.

Modal Order	Description	FAST Model Frequency (Hz)	Beam Model Frequency (Hz) (Error)	Multi-Scale Model Frequency (Hz) (Error)
1	1st side-side bending	0.32	0.32	0.32
			(0.00%)	(0.00%)
2	1st fore-aft bending	0.32	0.32	0.32
			(0.00%)	(0.00%)
3	2nd side-side bending	1.19	1.12	1.12
			(5.88%)	(5.88%)
4	2nd fore-aft bending	1.19	1.12	1.12
			(5.88%)	(5.88%)
5	1st torsional	3.44	3.43	3.44
			(0.29%)	(0.00%)
6	3rd side-side bending	4.12	3.75	3.76
			(8.98%)	(8.74%)
7	3rd fore-aft bending	4.12	3.87	3.88
			(6.07%)	(5.83%)
8	1st extensional	5.34	5.42	5.43
			(0.15%)	(0.17%)

**Table 4:** The basic material constants for the joint of jacket-type offshore wind turbine.

Fatigue Strength		Slope		Fatigue Constant		Stress Ratio
**Axial,** $s_{axi}$	**Torsion,** $s_{tor}$	**Axial,** $k_{axi}$	**Torsion,** $k_{axi}$	**Axial,** $C_{axi}$	**Torsion,** $C_{tor}$	−1
52	$s_{axi}/\sqrt{3}$	5	3	5.335 × 10^15^	3.705 × 10^11^	

**Table 5:** Wind and wave load calculation parameters.

50-Year Recurrence Period Load	Value
Mean wind speed (m/s)	42.7
Significant wave height (m)	9.4
Spectral peak period (m)	10.9

**Table 6:** Comparison of stress response characteristics between beam model and multi-scale model.

	$s_{m}$ (MPa)	$\sigma_{x}$ (MPa)	$S_{k}$	$K_{u}$	*m*	*C*	*E*[*D*]
Beam	35.527	4.313	0.278	3.813	5	5.335 × 10^15^	2.13 × 10^−10^
Multi-scale	36.987	4.495	0.249	3.748	5	5.335 × 10^15^	2.51 × 10^−10^
error	3.95%	4.06%	11.99%	1.74%	-	-	15.14%

**Table 7:** Uniaxial/multiaxial fatigue analysis parameters and results at P2.

	*m*	*K*	*C*	*E*[*D*]	ε
Multi-scale $\sigma_{xx}$	1	5	5.34 × 10^15^	2.51 × 10^−10^	-
Multi-scale $\sigma_{eq}$	2.03	7.06	5.84 × 10^18^	2.80 × 10^−10^	10.40
Multi-scale $\sigma_{eq}^{*}$	1.72	6.43	5.57 × 10^17^	2.92 × 10^−10^	14.05

**Table 8:** Uniaxial/multiaxial fatigue analysis parameters and results at P4.

	*m*	*K*	*C*	*E*[*D*]	ε
$\mathrm{Multi}-\mathrm{scale}\;\sigma_{xx}$	1	5	5.34 × 10^15^	1.31 × 10^−11^	-
$\mathrm{Multi}-\mathrm{scale}\;\sigma_{eq}$	1.41	5.82	5.81 × 10^16^	1.40 × 10^−11^	6.28
$\mathrm{Multi}-\mathrm{scale}\;\sigma_{eq}^{*}$	1.43	5.85	6.51 × 10^16^	1.49 × 10^−11^	13.29

**Table 9:** Uniaxial/multiaxial fatigue analysis parameters and results at P7.

	*m*	*K*	*C*	*E*[*D*]	ε
$\mathrm{Multi}-\mathrm{scale}\;\sigma_{xx}$	1	5	5.34 × 10^15^	2.87 × 10^−11^	-
$\mathrm{Multi}-\mathrm{scale}\;\sigma_{eq}$	1.85	6.70	1.51 × 10^18^	1.11 × 10^−11^	74.20
$\mathrm{Multi}-\mathrm{scale}\;\sigma_{eq}^{*}$	1.72	6.45	5.99 × 10^17^	1.67 × 10^−11^	82.25

## Data Availability

Not applicable.
